# Caloric restriction-mimetics for the reduction of heart failure risk in aging heart: with consideration of gender-related differences

**DOI:** 10.1186/s40779-022-00389-w

**Published:** 2022-07-04

**Authors:** Lei Pang, Xi Jiang, Xin Lian, Jie Chen, Er-Fei Song, Lei-Gang Jin, Zheng-Yuan Xia, Hai-Chun Ma, Yin Cai

**Affiliations:** 1 Department of Anesthesiology, the First Hospital of Jilin University, Changchun, 130021 China; 2 Health Promotion Center, the First Hospital of Jilin University, Changchun, 130021 China; 3 Department of Urology, the First Hospital of Jilin University, Changchun, 130021 China; 4 Henry Fok School of Biology and Agriculture, Shaoguan University, Shaoguan, 512000 Guangdong China; 5 Department of Metabolic and Bariatric Surgery, Jinan University First Affiliated Hospital, Guangzhou, 510630 China; 6 Department of Medicine, LKS Faculty of Medicine, the University of Hong Kong, Hong Kong, China; 7 State Key Laboratory of Pharmaceutical Biotechnology, LKS Faculty of Medicine, the University of Hong Kong, Hong Kong, China; 8 Department of Anesthesiology, Affiliated Hospital of Guangdong Medical University, Zhanjiang, 524000 Guangdong China; 9 Department of Health Technology and Informatics, the Hong Kong Polytechnic University, Hong Kong, China

**Keywords:** Cardiovascular disease, Cardiac aging, Caloric restriction, Gender difference, Caloric restriction-mimetics, Dietary compounds, Clinical application

## Abstract

The literature is full of claims regarding the consumption of polyphenol or polyamine-rich foods that offer some protection from developing cardiovascular disease (CVD). This is achieved by preventing cardiac hypertrophy and protecting blood vessels through improving the function of endothelium. However, do these interventions work in the aged human hearts? Cardiac aging is accompanied by an increase in left ventricular hypertrophy, along with diastolic and systolic dysfunction. It also confers significant cardiovascular risks for both sexes. The incidence and prevalence of CVD increase sharply at an earlier age in men than women. Furthermore, the patterns of heart failure differ between sexes, as do the lifetime risk factors. Do caloric restriction (CR)-mimetics, rich in polyphenol or polyamine, delay or reverse cardiac aging equally in both men and women? This review will discuss three areas: (1) mechanisms underlying age-related cardiac remodeling; (2) gender-related differences and potential mechanisms underlying diminished cardiac response in older men and women; (3) we select a few polyphenol or polyamine rich compounds as the CR-mimetics, such as resveratrol, quercetin, curcumin, epigallocatechin gallate and spermidine, due to their capability to extend health-span and induce autophagy. We outline their abilities and issues on retarding aging in animal hearts and preventing CVD in humans. We discuss the confounding factors that should be considered for developing therapeutic strategies against cardiac aging in humans.

## Background

Cardiac aging is a natural process and is accompanied by the progressive development of cardiac hypertrophy and dysfunction [1, 2]. As a major contributor to cardiovascular disease (CVD), cardiac aging occurs in both sexes with most of the burden falling on middle-aged and older adults. This is because cardiac aging predisposes the heart to stress, thereby increasing cardiovascular mortality in the elderly [3, 4]. Incidence and prevalence of age-related CVD, such as hypertension, atherosclerosis, coronary, and cerebral artery disease increase dramatically in men aged around 45, and 10 years later in women who reach menopause [5]. A sharp increase is also evident in post-menopausal women [6]. Females are usually under-represented in clinical trials, as the participants in most studies evaluating CVD risk factors are men instead of mixed populations [7, 8]. Evidence shows that, besides age, comorbidities and comedications, as well as additional confounding factors such as sex hormones may affect the endogenous cardioprotective aspects. Thus, there is an unmet need to assess whether gender differences in age-related comorbidities associated with heart failure (HF) require specific management strategies.

**Fig. 1:**
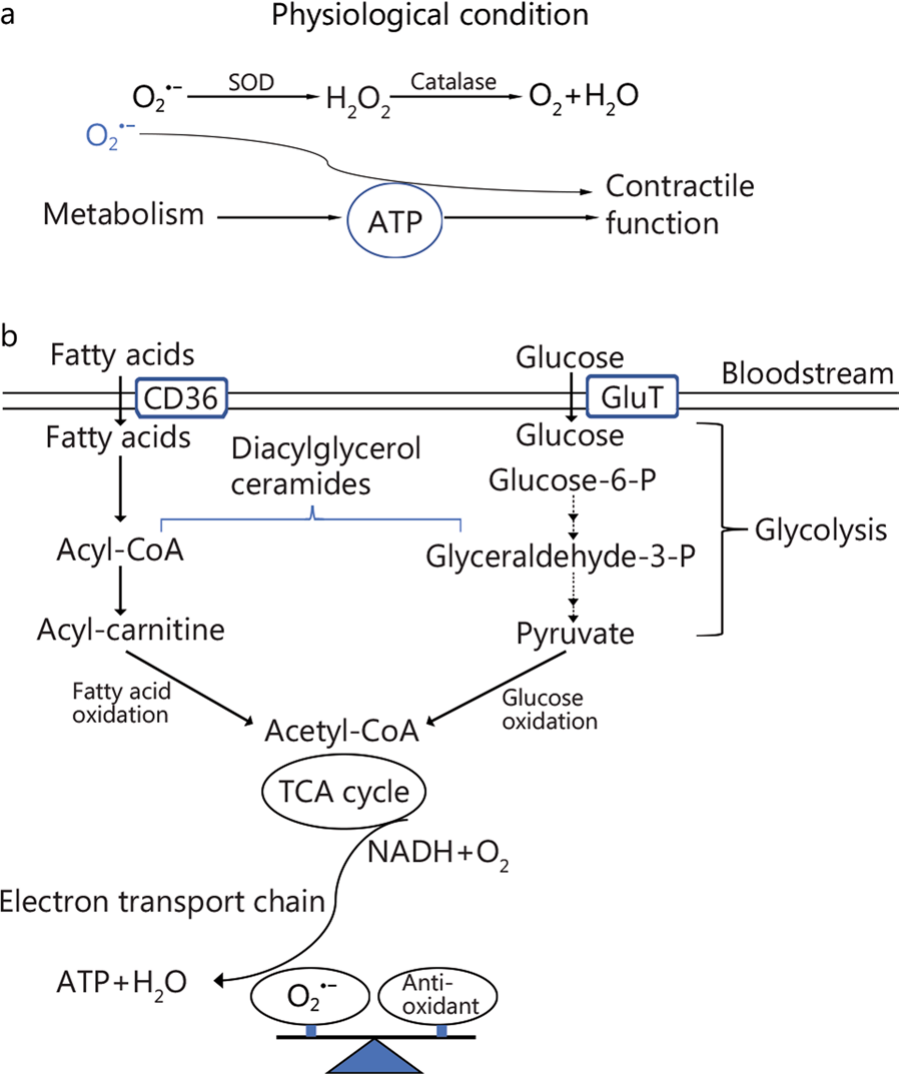
Energy metabolism in the heart under physiological condition. **a** In a normal heart, the main task of energy metabolism is to produce ATP for the pumping function. **b** To maintain a high energy demand, the heart is equipped with an enzymatic machinery orchestrating ATP production that mainly uses fatty acids and glucose under physiological condition. Under physiological condition, the production of reactive species is minor, and mainly produced in the mitochondria with superoxide (${\mathrm{O}}_{2}^{ \cdot -}$) as the primary form in the heart. Of which, the balance is maintained by the action of superoxide dismutase and catalase by converting them to O_2_ and H_2_O. ATP adenosine triphosphate, CD36 cluster of differentiation 36, SOD superoxide dismutase, TCA tricarboxylic acid

**Fig. 2:**
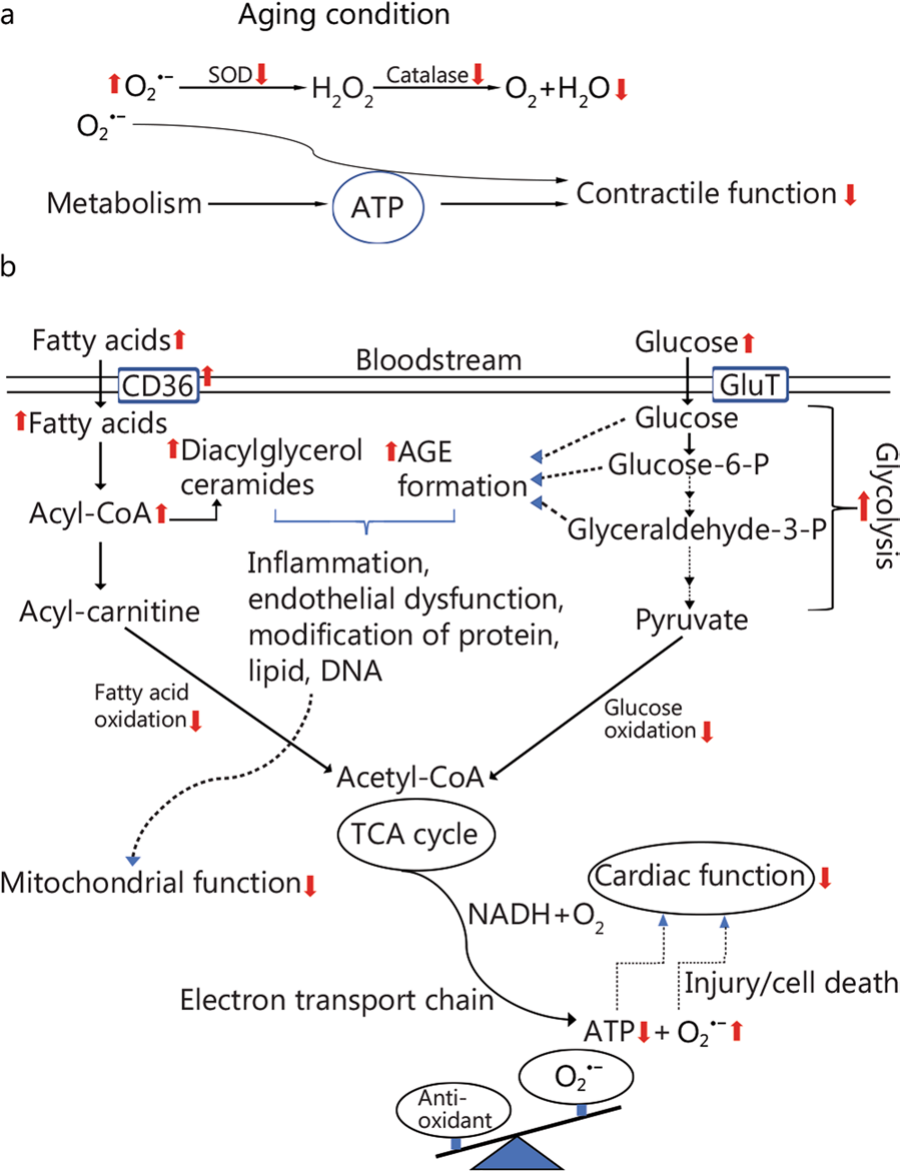
Metabolic alterations in the aged heart. **a** Under an aging condition, ATP production is reduced. **b** Although the aged heart takes up more lipid, myocardial fatty acid oxidation is reduced concomitant with an accumulation of lipids. In parallel, glycolysis is uncoupled from glucose oxidation, leading to an accumulation of advanced glycation end-products (AGE), as a by-product of glycolysis, which, together with accumulated myocardial intralipids, promotes inflammation and alters intracellular redox condition, as well as the modification of protein, lipid, and DNA. As a result, mitochondrial dysfunction and the formation of reactive oxygen species, such as ${\mathrm{O}}_{2 }^{ \cdot -}$, exceeds the antioxidative capacity, resulting in endothelial dysfunction, cell injury, and cardiac dysfunction. ATP adenosine triphosphate, CD36 cluster of differentiation 36, SOD superoxide dismutase, TCA tricarboxylic acid

Since the postulation of free radical theory of aging, mitochondrial theory has been a key focus area for aging research. Mitochondrial theory focuses on mitochondria as the main producer of reactive oxygen species (ROS) [9], while radical theory focuses on ROS as the effector of oxidative stress [10], which is beyond the threshold of an endogenous antioxidant system [11]. ROS is produced along the electron transport chain [12], in which electrons are capable of establishing a proton gradient that is necessary for ATP production and is completely neutralized with oxygen to water (Fig. 1a). Both theories suggest that the cellular deterioration seen with increasing age is related to mitochondrial dysfunction, causing endothelial dysfunction, alteration in the vasculature, and/or vascular injury [11, 12]. Given the extraordinary demand for energy, the heart contains the highest density of mitochondria, allowing it to produce cellular adenosine triphosphate (ATP) mainly from fatty acid oxidation (Fig. 1b), with glucose metabolism contributing less [13]. Myocardial ATP production and fatty acid oxidation decline in the aged human heart, concomitant with the accumulation of lipids [14] (Fig. 2a). Meanwhile, increased activity of myocardial aldose reductase and sorbitol dehydrogenase in the aged heart enhances polyol pathway by driving the flux of glucose to sorbitol [15]. This alters not only intracellular redox status by decreasing the synthesis of reduced glutathione and nitric oxide (NO) production, but also the modification of protein, lipid, and DNA with advanced glycation endo-products (Fig. 2b) to promote ROS production. Thus, alteration in mitochondrial metabolism in an aging heart is the underlying basis for the increased sensitivity to stress.

**Fig. 3:**
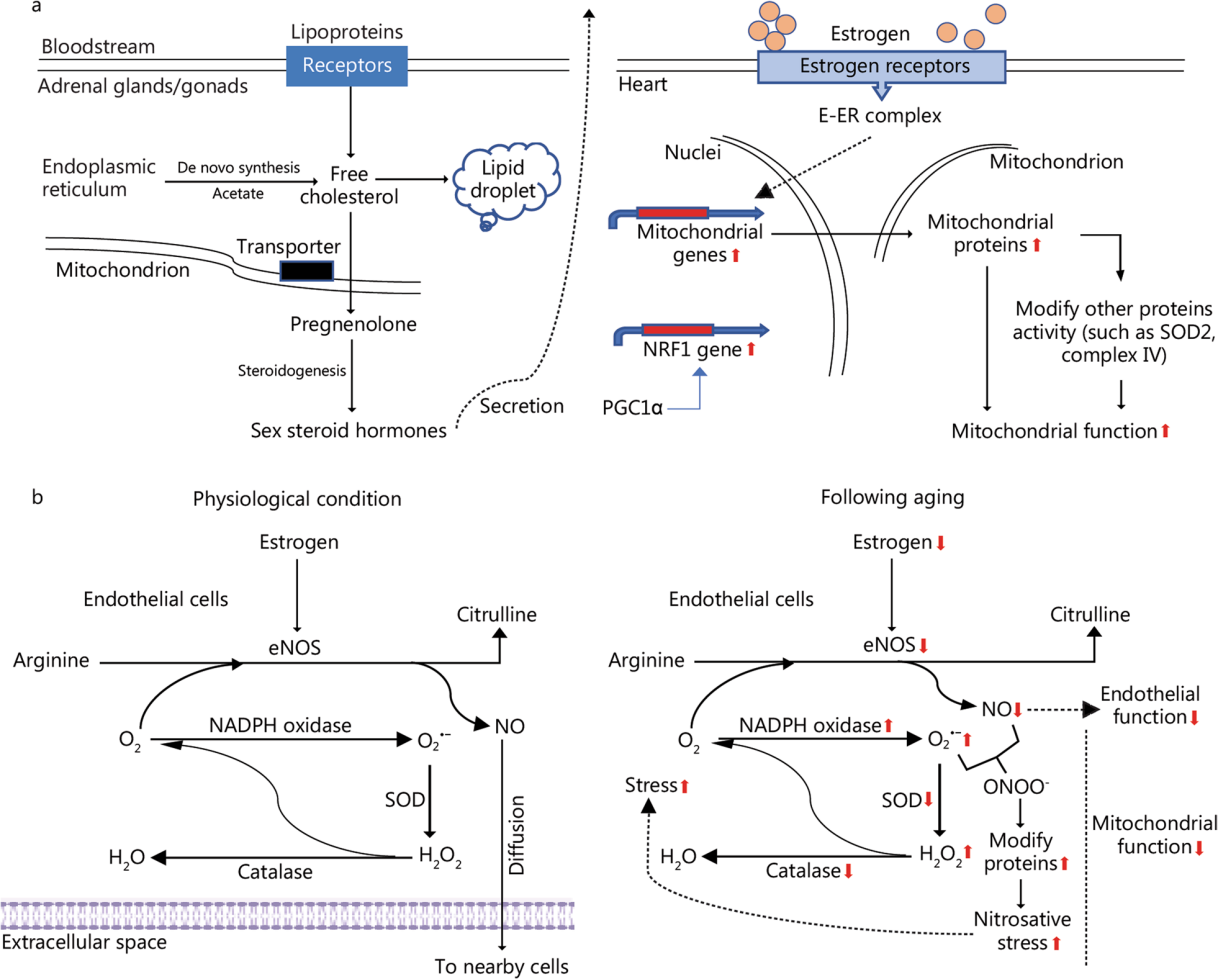
Interplay between sex hormones, mitochondrial function, and endothelial function. **a** All steroid hormones are made from cholesterol, which has two potential sources from either de novo synthesis by using acetate or importing of circulating high density lipoproteins (in rodent cells) and low-density lipoproteins (in human steroidogenic cells). Intracellular free cholesterol can be re-esterified and stored in lipid droplets or reach the outer mitochondrial membrane then move into inner mitochondrial membrane where it can be converted to pregnenolone as substrate for steroidogenesis. Mitochondrial integrity is important in the biosynthesis of sex steroid hormones by modulating enzymes for steroidogenesis and by maintaining cells that produce these hormones. After secretion, circulating estrogen (E) form a complex with estrogen receptor (ER) to exert its intracellular function through both genomic and non-genomic actions. For example, through modulating the gene of transcription factors, such as peroxisome proliferator-activated receptor gamma coactivator 1α (PGC1α) and nuclear respiratory factor-1 (NRF1) to control transcription of mitochondrial encoded genes or alter mitochondrial function by modification of mitochondrial proteins. **b** Estrogen is a primary target of endothelial nitric oxide synthase (eNOS), which converts arginine into citrulline along with the formation of nitric oxide (NO) in the process. Under normal physiological conditions, NO is the predominate product exhibiting positive cardiovascular effects. Following aging, estrogen deprivation is accompanied by a reduced eNOS activity, resulting in an accumulation of reactive oxygen species, thereby scavenging NO to reduce its bioavailability. As a result, stress related protein modification accelerates age-related arterial stiffening and endothelium dysfunction. SOD superoxide dismutase

The interplay between mitochondria function and sex steroid hormone biosynthesis (Fig. 3a) is evident [16]. Age-related decrease in sex hormone and mitochondrial dysfunction has been demonstrated in both men and women [17]. Emerging evidence has revealed that signaling pathways in the aged human hearts differ between males and females—specifically in the context of anti-oxidative defense, inflammation state, and mitochondrial biogenesis [18]. Additionally, failing human hearts with preserved ejection fraction (HFpEF) display a distinctive metabolic profile and gene transcriptome from that with reduced ejection fraction (HFrEF) [19]. Currently, there are several therapies for treating HFrEF, including devices, transplants and medications with beta‐blockers and sodium‐glucose co‐transporter 2 inhibitors. Of which, however, none have been shown to be effective for HFpEF in randomized clinical trials [20, 21].

In contrast, herbal or dietary compounds, rich in polyphenols or polyamine, have become alternative therapy with several advantages, such as being relatively inexpensive compared to pharmaceutical drugs [22]; relatively easy for most people to receive benefits through dietary modifications or supplementation and starting from an earlier age. More importantly, epidemiological studies have demonstrated that regular consumption of polyphenol-rich foods may reduce the risk of CVD and slow cardiac aging [23]. Hence, we have selected a few dietary compounds, including resveratrol, quercetin, epigallocatechin gallate (EGCG), curcumin, and spermidine, due to their capacity to extend health-span in model organisms, alleviate cardiac aging [24–35] (Table 1), and prevent CVDs in humans [36–61] (Table 2). As most of the studies regarding the beneficial effects of the above-mentioned compounds were carried out using genetically homogeneous laboratory strains, which, in contrast to genetic diversity in human populations [62], it remains unclear which of the effects would be beneficial for an aging human heart. Thus, this study will not present the extensive literature on signaling pathways of caloric restriction (CR)-mimetics in animal studies, and instead, will discuss their effect on preventing CVDs in human hearts (Table 2). We will focus on three areas: (1) cardiac remodeling and molecular and cellular mechanisms; (2) gender-related differences and potential mechanisms underlying diminished cardiovascular response in older men and women; (3) protective effect of the above-mentioned compounds on aged animal hearts and CVD in humans, as well as the confounding factors that should be considered for developing therapeutic approaches against cardiac aging in humans. We believe that understanding the differences of molecular mechanisms underlying the cardiac aging process in both males and females will lay a foundation for new therapeutic strategies that ensure effective gender-specific intervention strategies.

**Table 1:** Resveratrol, Quercetin, Curcumin, EGCG and Spermidine alleviates cardiac aging in animal models

CR-mimetics	Animal/dose	Duration	References
Resveratrol	2 months old mice, 4.9 mg/(kg·d)	8 months	[24]
	10 months old mice, 20 mg/(kg·d)	4 d	[25]
	14 months old mice, 50 mg/(kg·d)	16 months	[26]
Quercetin	1 year old diabetic Zucker Diabetic Fatty rats, 20 mg/(kg·d)	6 weeks	[27]
	8 weeks old dystrophin-deficient mice, diet with 0.2% quercetin	8 months	[28]
Curcumin	26–28 months old rats, 50 mg/(kg·d)	2 months	[29]
EGCG	16 months old mice, 50 mg/(kg·d)	8 weeks	[30]
	24–26 months old mice, 200 mg/(kg·d)	1 month	[31]
Spermidine	4 months old mice, 3 mmol/L in drinking water	26 months	[32]
	18 months old mice, 3 mmol/L in drinking water	6 months	[33]
	27–29 months old mice, 3 mmol/L in drinking water	4 weeks	[34]
	7 weeks old Dahl salt-sensitive rats, 3 mmol/L in drinking water	12 weeks	[32]
	22–24 months old rats, 10 mg/(kg·d)	6 weeks	[35]

**Table 2:** Resveratrol, Quercetin, Curcumin, EGCG and Spermidine prevents CVD in randomized clinical trials with patients

CR-mimetics	Patients/dose	Duration	References
Resveratrol	Type 2 diabetic patients, 10 mg/d (*n* = 10) or 250 mg/d (*n* = 28), respectively	1 and 3 months	[36, 37]
	Patients with stable angina pectoris, 20 mg/d (*n* = 29) or 100 mg/d (*n* = 30), respectively	2 months	[38, 39]
	Patients post ischemia infarction, 10 mg/d (*n* = 20), or patients with HFrEF (*n* = 30), 100 mg/d	3 months	[40, 41]
	Patients with high risk of CVD (*n* = 75); 8 mg/d or 16 mg/d for 6 months	12 months	[42]
	Hypertensive patients (*n* = 24), 300 mg or obese men and post-menopausal woman (*n* = 19), 30–270 mg	Single dose	[43, 44]
	Obese individual (*n* = 28), 75 mg/d	6 weeks	[45]
	Obese men (*n* = 11), 150 mg/d	4 weeks	[46]
	Patients with metabolic syndrome (*n* = 22); 2 pills FRAMINTROL (contains 100 mg/d resveratrol)	3 months	[47]
Quercetin	Both gender (*n* = 37), quercetin-3-glucoside (160 mg/d)	4 weeks	[48]
	Women with type 2 diabetes (*n* = 72), 500 mg/d	10 weeks	[49]
	Patients with stable coronary heart disease (*n* = 30), 120 mg/d	2 months	[50]
	Overweight patients, 150 mg/d (*n* = 93), or 163 mg/d (*n* = 70) quercetin from onion skin extract	5 and 6 weeks	[51, 52]
	Pre-hypertensive men and women (*n* = 37), 160 mg/d quercetin-3-glucoside	4 weeks	[48]
Curcumin	Patients with coronary artery bypass grafting (*n* = 121), curcuminoids 4 g/d	9 d	[53]
	Type 2 diabetes, 1000 mg/d (*n* = 30), or 300 mg/d (*n* = 50), respectively	12 weeks	[54, 55]
	Postmenopausal women, 150 mg/d (*n* = 32), or 150 mg/d (*n* = 45), respectively	8 weeks	[56, 57]
EGCG	Patients with coronary artery disease (*n* = 42), 300 mg/d	2 weeks	[58]
	Patients with early atherosclerosis (*n* = 28), 30 ml of EGCG containing olive oil/day	4 months	[59]
	Obese postmenopausal women (*n* = 38), 300 mg/d	12 weeks	[60]
Spermidine	Healthy male volunteers (*n* = 30), aged from 40 to 69 years, with Japanese food (natto)	12 months	[61]

## Mechanisms underlying age-related cardiac remodeling

### Remodeling in aged heart

This refers to a series of changes related to cardiac aging and vascular aging at the cellular and molecular levels [63]. The structural and functional transformation of a human heart occurs in healthy adults aged between 20 to 85 years old [64]. Structurally, it mostly affects blood vessel geometry, valves, and chambers, such as thickening of blood vessels and heart valves, increasing the size and volume of the left atrium, left ventricle (LV) hypertrophy, and interventricular septum, accompanied by increased wall thickness and interstitial fibrosis [63]. Functionally, diastolic function declines with advanced age, in both LV and the right ventricle, which can be assessed by diastolic filling in two phases: passive filling (early) and active filling (late) [65]. The rates of early diastolic filling in LV progressively decline after age 20 and reduce up to 50% by age 80 with increased filling in late diastole [66]. These changes are similar to the filling profile in the right ventricle [67]. Although systolic function with respect to ejection fraction is not affected at rest [4], age-related ventricular dysfunction is evident under exercise [68].

### Intracellular processes in aged heart

Arterial stiffening and endothelial dysfunction are both characteristics of vascular aging. The pathways can be quite diverse, but attributable to a prolonged imbalance between damaging and repairing [69].

On a cellular level, the composition of a mammalian heart is often described in the context of cardiomyocytes and non-cardiomyocytes. Cardiomyocytes are the contractile element, contributing to 35% of the total cells in the myocardium [70, 71]. Non-cardiomyocytes include a diverse set of cells, such as fibroblasts and endothelial cells. The first line of evidence regarding an imbalance in damaging and repairing is the reduced regenerative capacity of the heart, which relies not only on proliferation of cardiomyocytes, but also on populations of other cells. In this case, there are two major challenges. One is the demise of cells due to necrosis and apoptosis [72, 73]. Apoptosis occurs not only on cardiomyocytes, but also on endothelial cells, in response to age-related alterations in systemic and local environment, as well as cell–cell communication impairment [74]. Another challenge is the low regenerative capacity of the heart because adult cardiomyocytes are terminally differentiated cells, and the aging heart contains more senescent cardiomyocytes [1]. The regenerative capacity of a murine heart is reduced from day 7 post-birth, while the mitotic activity of cardiomyocytes is lost during adulthood, largely due to age-related increase in the number of fibroblasts [75, 76]. Likewise, the regeneration rate of human cardiomyocytes is approximately 1% annually in young adults, which diminishes to 0.45% in the elderly [77], indicative of limited regenerative capacity in an adult human heart. Another line of evidence regarding imbalance theory in an aging heart is the impaired dynamic crosstalk between cardiomyocytes and non-cardiomyocytes, such as endothelial cells [76, 78, 79]. To this point, one form of direct evidence is demonstrated via vascular endothelial growth factor (VEGF), an endothelial cell marker protein, which functions via its receptor (VEGF-receptor) on the surface of cardiomyocytes. Adult mice with deletion of VEGF-receptor displayed an increase in the coronary vasculature and induction of cardiomyocytes hypertrophy [80]. Additionally, mice with deletion of apelin, a protein produced by endothelial cells [81], developed a progressive impairment of cardiac contractility associated with systolic dysfunction [82]. Furthermore, miR-217 is identified as the most highly inducible miRNA during human endothelial cell aging [83]. Mice with overexpression of endothelial-specific miR-217 displayed endothelial dysfunction in conjunction with an altered left ventricular diastolic and systolic dysfunction [83].

On a molecular level, multiple factors contribute to the damaging mechanism of an aging heart, such as autophagy (an intracellular recycling program targets dysfunctional organelles and proteins to lysosomes for degradation), mitochondrial dysfunction, oxidative stress, inflammation, and genomic instability caused by DNA damage or telomere attrition [84, 85]. A significant challenge is to dissect their relative contribution to aging due to interrelation. However, mitochondrion plays a critical role in forming the crossroads for the pathways related to cardiac aging [86]. Mitochondrial function is determined by mitochondrial dynamics, which includes a network process of mitochondrial fusion, fission, and biogenesis, in which mitophagy, a specific form of autophagy to remove dysfunctional mitochondria, is essential for mitochondrial morphology, quality and abundance [87]. Mitophagy activity is downregulated during aging, concomitant with a decline in mitochondrial function [32], and increased ROS generation in aged cardiomyocytes [88]. This is associated with functional impairment at the organ levels, such as diastolic dysfunction, LV hypertrophy, increased risk of atrial fibrillation, and decreased exercise capacity in an aging heart [63]. On a mechanistic level, ROS-induced DNA damage is a key regulator of autophagy in aging heart. In adult cardiomyocytes, more chemical energy is consumed by excitation and contraction than in other non-contractile cells, so the adult heart greatly relies on cellular quality control mechanisms to maintain mitochondrial quality. However, ROS progressively accumulate during aging, which induces mutations in mitochondrial DNA and impedes the tricarboxylic acid cycle and electron transport chain complexes, thereby progressively reducing mitochondrial DNA content and promoting mitochondrial dysfunction [89]. That said, signaling through oxidative stress/DNA damage axis also promotes redox-sensitive mediators, such as nuclear factor κB, that, in turn, modulates the transcription of several pro-inflammatory cytokines [90]. It is evident that targeting mitochondria-inflammation circuit can mitigate HFpEF [91]. Direct evidence of oxidative stress-linked mitochondrial dysfunction is provided by using mice overexpressing mitochondrial-targeted catalase, which is an antioxidant enzyme [92]. Mitochondrial-targeted catalase prevents ROS-mediated damage on mitochondrial DNA and increases median life span [92]. Furthermore, telomere dysfunction links cardiac dysfunction through telomere-p53/peroxisome proliferator-activated receptor gamma coactivator 1-alpha (PGC1α)/mitochondrial axis along with the changes, such as decreased ATP generation capacity and increased oxidative stress, seen in aged mouse heart [1, 93]. Collectively, mitochondrion is a primary place of ROS production, which in turn leads to a forward-feedback spiral of increasing damage to mitochondrial DNA, as reflected in the aged heart of laboratory animals to humans [94]. Thus, mitochondrial dysfunction is a major contributor to heart senescence, irrespective of the differences between individuals and species [95].

### Interplay between sex steroid hormones, mitochondrial function and endothelial function

Evidence suggests that sex hormone deficiency contributes to oxidative stress in the aged heart [96]. Estrogen, progesterone, and testosterone are sex steroid hormones and are classically functional by binding to their receptors [96]. All steroid hormones are made from cholesterol, which has two potential sources, either de novo synthesis by using acetate or importing circulating lipoproteins (Fig. 3a). Intracellular free cholesterol can be stored in lipid droplets or moved into inner mitochondrial membrane where it can be converted to pregnenolone as a substrate for steroidogenesis. Importantly, mitochondria modulate the enzymes for steroidogenesis. 17beta-estradiol is the major female sex hormone of circulating estrogen, which is functional through forming a complex with its receptor, followed by translocation into the nucleus for directly binding to DNA sites known as estrogen response elements or indirectly binding to DNA through other transcriptional factors (Fig. 3a) [97].

In particular, estrogen can regulate endothelial function through enzymes related to mitochondrial oxidative stress. One line of evidence is that estrogen up-regulates not only factors associated with mitochondrial biogenesis, electron transport chain, and complex IV activity for enhancing mitochondrial function, but also superoxide dismutase (SOD) activity for reducing cellular levels of ROS [98]. The age-dependent decrease of mitochondrial SOD2 has been observed in female human hearts, but not in male hearts [18], suggesting its favorable expression in younger female hearts. Meanwhile, estrogen also regulates endothelial NO synthase (eNOS) (Fig. 3b), which can be two completely different physiological outcomes depending on eNOS as a source of NO or superoxide [99]. Under normal conditions in younger women, the production of NO in the vasculature is generally considered to have positive cardiovascular effects [100]. As a woman ages, estrogen deprivation is accompanied by a reduced eNOS activity and mitochondrial dysfunction (Fig. 3b), resulting in reduced NO bioavailability and ROS accumulation along with arterial stiffening and decline of endothelium-dependent vasodilation [101], in postmenopausal women than in young women [102]. Of which, the reduction is more pronounced in late perimenopausal women than early perimenopausal women, despite being similar in age [103]. The flow-mediated vasodilation is increased in estrogen-deficient postmenopausal women with systemic infusion of ascorbic acid, a nonspecific antioxidant, whereas the effect is not observed with estradiol [104]. Thus, oxidative stress and endothelial dysfunction has a positive association in estrogen-deficient postmenopausal women [105, 106].

## Gender-related differences in aged heart

Accumulating evidence has demonstrated that men are at higher risk of CVD than women, with sex steroids playing a role [107]. Clinical trials have demonstrated that women living with HF, despite being older, have significantly better survival rates than men [108]. This is attributable to gender-related differences with respect to human LV morphology, chamber function, hemodynamics as well as changes of sex hormone and mitochondrial function with aging [109].

### Cardiac aging—LV mass and chamber function

Cardiac aging is accompanied with the development of cardiac hypertrophy. LV mass is lower while LV contractility is stronger in women relative to men [7], which is attributed to a lower apoptotic rate of myocytes in women, thereby preserving LV mass with aging [7]. The maximal cardiac pumping capability is preserved with age in women, but decreases by 20–25% in men [110]. Likewise, age-related cardiac dysfunction, as reflected by the maximal aerobic capacity or end diastolic volume response to vigorous exercise, is more pronounced in men than women of similar ages. This difference becomes blunted in women across age range [111].

### Vascular aging—hemodynamics, arterial function, and endothelial function

Women have a faster heart rate, lower blood pressure, and a higher index of LV afterload than men at all comparable ages [112]. Age-related abnormalities in systemic arteries occur earlier in men than in women, irrespective of the known risk factors [113]. Likewise, levels of estrogenic hormone affect arterial distensibility, as reflected by stiffer pre‐pubertal arteries and distensible post‐puberty arteries in women than their male counterparts [112]. Pulse pressure is an accepted predictor of CVD in human populations, and younger women have a lower pulse pressure [112]. Thus, the risk of CVD is much lower in pre‐menopausal women.

With respect to endothelial dysfunction: it starts 10 years earlier in healthy men than in women, who reach the time of menopause, indicating that endothelial function is, in part, controlled by sex steroid-dependent pathways [6]. Indeed, a steep decline of endothelial function is more evident in elderly women than in men [6]. Excessive circulating androgen may be related to endothelial dysfunction and arterial stiffness in menopausal women, as reflected by coronary artery calcification [114, 115] and carotid intima media thickness [116]. This notion, however, needs further evidence to corroborate. As an important androgen in males, circulating testosterone concentrations decline as men age [117]. Testosterone replacement strategy can mitigate some of the aging characteristics, but it does not significantly improve physical function in aged men [118, 119]. The effect of testosterone on the cardiovascular system ranges widely from being protective to deleterious and even no association at all in men [120, 121]. In addition, an increased risk of developing symptomatic coronary heart disease has been reported in postmenopausal women with the lowest and highest quintile of bioavailable testosterone compared with women in the middle quintile [122]. Obviously, we are far from concluding that estrogens are cardioprotective and androgens are detrimental.

### Phenotypes of HF and lifetime risk factors

**Fig. 4:**
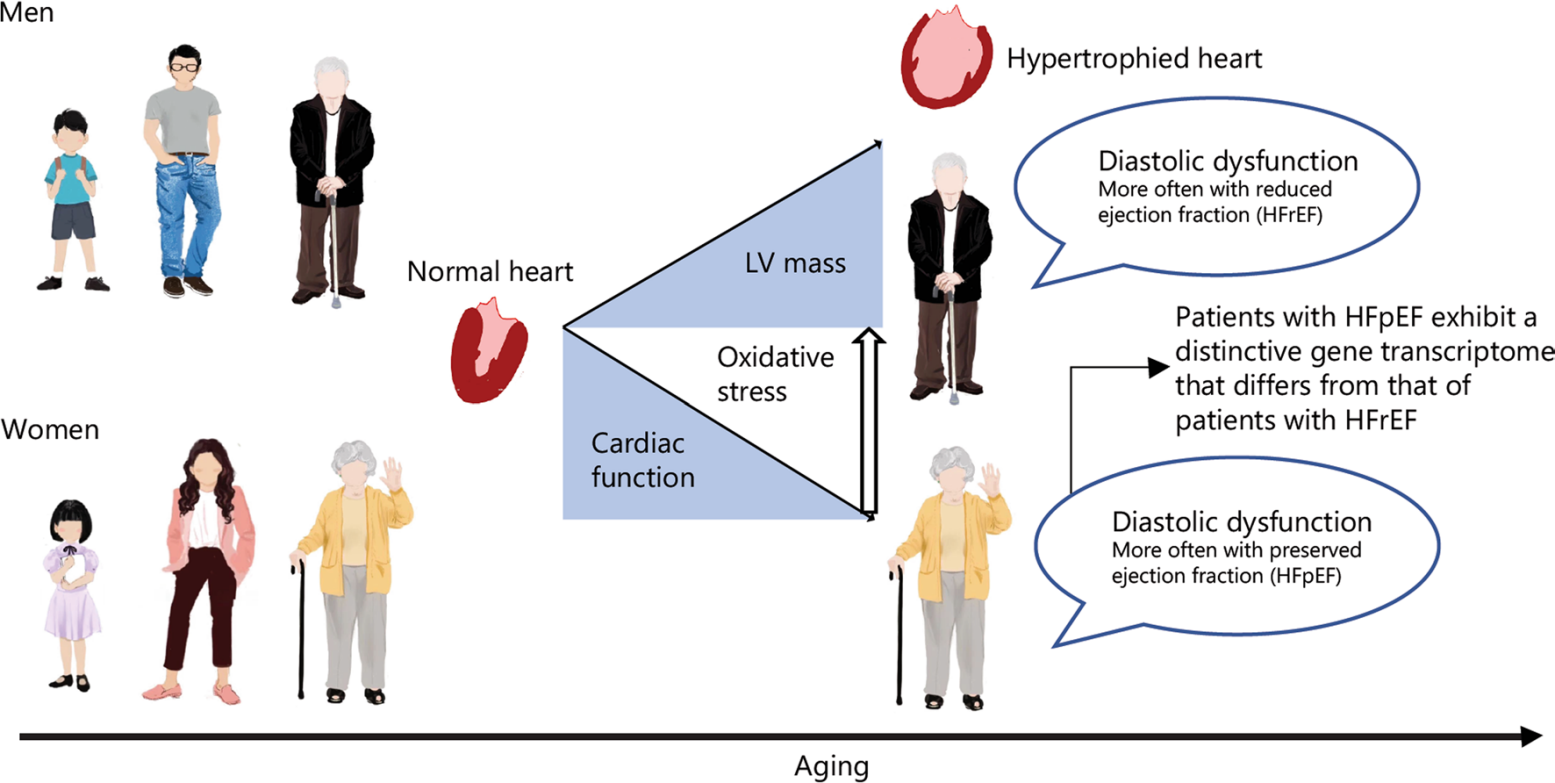
Gender-related differences in the phenotypes of heart failure. Heart failure occurs during aging in both men and women with increased left ventricular (LV) mass and reduced cardiac function. Female is often associated with diastolic dysfunction and preserved ejection fraction (HFpEF) as opposite to male, which is mainly associated with diastolic dysfunction but reduced ejection fraction (HFrEF). Patients with HFpEF exhibit a distinctive gene transcriptome that differs from that of patients with HFrEF in systemic inflammation, cardiac remodeling, and stiffness. LV left ventricle

Age-related HF can be HFpEF or HFrEF (Fig. 4), in association with gender and races. Most older women have HFpEF in contrast to HFrEF in men [123]. Of interest, the highest proportion of hospitalized patients with HFpEF is white women (59%), in contrast to black males in HFrEF (70%) [124]. Regarding lifetime risk factors: ischemic heart disease is the etiology frequently causing HFrEF in men, whereas HFpEF is a multifactorial disease with obesity, diabetes, valve disease, and hypertension, which is predominant in women [7]. Although lifetime risks of developing HF are equal between men and women by the age of 40 [125], the incidence is higher in men, while the prevalence is higher in women with advancing age [126]. However, the rates of HF for women aged between 65–84 triples with each decade, which, only double in men [7, 125]. Of interest, the lifetime risks for developing HF are higher in white males and females relative to their black counterparts [127]. Regarding prognosis: both gender-related and gender-unrelated relationships have been described in the literature. Among hospitalized patients with HFpEF, 20% lower mortality rates are found in women relative to men [128]. In contrast, recent studies have shown that age and creatinine are the only variables associated with mortality rate since the probability of survival does not show gender-related difference between patients admitted for decompensated HF [8, 129].

## CR-mimetics in aged heart

### Health concerns of CR

The principle of CR is to achieve a weight loss with a life-long reduction (30–40%) of caloric intake by using a diet containing adequate amounts of protein, vitamins and minerals [130]. As expected, data regarding the long-term effect of CR in humans is scarce. One of the reasons may be due to the difficulties of adhering to the life-long rigorous intervention, which may explain some failures in the clinical studies [131]. Apart from loss of muscle mass and bone density in older women [132, 133], CR-related physiological symptoms also include hypotension, eating disorders, higher risk of hypothermia, loss of strength and stamina, slower wound healing, as well as a decline in sex steroids and infertility or preterm delivery in females [134–138]. Therefore, CR is not recommended for women prior to or during pregnancy [139]. Furthermore, CR-related psychological conditions include depression, emotional deadening, and irritability, which affect not only the quality of their life but also the family members who care for them [140, 141].

### Potential molecular mechanism of CR-mimetics in heart

As discussed above, mitochondrial dysfunction plays a critical role in cardiac aging. Thus, the efficient removal of defective mitochondria through autophagy is essential for maintaining viability and homeostasis of cardiomyocytes. The efficiency of autophagy declines gradually with age, which contributes to heart senescence and age-related CVD [142–144]. CR is an effective autophagy inducer. Hence, compounds that are considered as CR-mimetics, in addition to extending health-span [145], should have the capability of inducing autophagy [146, 147] by regulating intracellular levels of nicotinamide adenine dinucleotide (NAD^+^) through inhibiting acetyltransferases or activating deacetylases [145]. NAD^+^ levels decline with age [148, 149], and the plasma NAD^+^/NADH ratio is sex-dependent and higher in women than in men although the difference reduces with increasing age [150]. Notably, resveratrol [151] and quercetin [152] act as a deacetylase activator, while curcumin [153], EGCG [154], and spermidine [155] are well-established acetyltransferase inhibitors. They display cardio-protection in the aged animal hearts (Table 1) through distinct signaling pathways converging on the acetylproteome in association with autophagy.

## What is known about their effect on cardiac aging and CVD?

### Resveratrol

#### Sources

The highest content of resveratrol has been demonstrated as 30 mg/kg fresh weight of lingonberry (*Vaccinium vitis-idaea* L.) [156], followed by a higher content in dried grape skins or red grapes, relative to grapes themselves or white ones, respectively [157, 158].

#### Alleviating cardiac aging

The evidence that resveratrol can alleviate cardiac aging has been demonstrated in mice [24–26]. The mechanisms include restoring the expression of aging related transcription genes, such as Wnt/β-catenin signaling pathway, in the aged heart to levels found in a young heart [26] or protecting aged mice from myocardial toxicity [25] in a sirtuin 1 (SIRT1)-dependent manner [24]. With respect to cardiac aging in humans, the effect is unproven. However, it is evident that resveratrol augments autophagy to reverse remodeling in a post-infarction mouse heart [159], and shows great ability to reduce red blood cell aggregation in patients with HF [160]. All of these aspects may positively affect cell viability and microcirculation thereby improving oxygen supply in an aging heart.

#### Alleviating CVD

The effects of resveratrol on mitigating CVD risk factors have been attributed to its antioxidant [106], anti-inflammatory [161], antiplatelet [162], and lipid-lowering properties in preclinical studies [163, 164]. Regarding the effect in humans, the first clinical trial of resveratrol with CVD patients was reported by Brasnyó et al. [36]*,* in which, type 2 diabetic patients showed improvements in insulin sensitivity and oxidative stress after taking 10 mg of resveratrol daily for one month. Likewise, type 2 diabetic patients showed significant improvement in fasting blood glucose, systolic and diastolic blood pressure, and blood lipid profile with hypoglycemic drugs plus 250 mg daily resveratrol for three months when compared to hypoglycemic drugs alone [37]. In addition, the cardioprotective effect of resveratrol in humans are evident in patients with ischemic heart disease or HF. Resveratrol was used for patients from 2 to 3 months with a dosage of 10–20 mg/d [38, 40] to 100 mg/d [39, 41]. Longer duration of treatment was one year, with 8 mg/d and 16 mg/d for each 6 months, respectively [42]. Although the patients either had myocardial infarction [40], stable angina pectoris [38, 42], coronary artery disease [39], or HFrEF [41], the effect of resveratrol on improving clinical parameters were mainly converged to improved endothelial function [40], cardiac function [39, 41], and reduced inflammation [42]. It is noteworthy that resveratrol increases the expression of glucose transporter 4 through activation of endothelial estrogen receptors (ERs) to improve mitochondrial energy metabolism [165]. This could be beneficial for postmenopausal women, as menopause has adverse effects on lipid and glucose metabolism [166, 167]. Currently, hormone therapy is not recommended for primary or secondary prevention of CVD at any age [168]. Therefore, resveratrol has been proposed to be a substitute of estradiol in women based on its effect on increasing endothelial function by enhancing the bioavailability of NO [169], as determined by using brachial artery flow-mediated vasodilation in healthy obese adults with or without mild hypertension, or in hypertensive postmenopausal women [43–45]. Importantly, resveratrol displays a beneficial effect on cognitive performance and total well-being in postmenopausal women [170], which can be sustained with ongoing supplementation for over a year [171].

#### Conundrum

Long-term studies are required to investigate the efficacy of resveratrol in humans, especially in patients with CVD. The overall beneficial effects of resveratrol are not proportional to its dosing in humans. As an example, resveratrol supplementation for 30 d reduced insulin resistance [36, 46] or plasma triglyceride [46] concentration at both low (10 mg/d) and moderate doses (150 mg/d), but not at high doses (1–2 g/d) [172]. The bioavailability of resveratrol is not reported in these studies but could be a potential reason attributable to the conflicting results. In general, oral resveratrol has low bioavailability but high absorption, due to a rapid hepatic first-pass metabolism [173]. Of note, the bioavailability of resveratrol can be greatly enhanced over 10 times by piperine, which is a type of bioactive alkaloid [174]. The intake of resveratrol appears to be at least 70% in the urinary excretion [175]. It is the sulfate conjugated form, rather than free form, of resveratrol detectable in human plasma samples within 2 h after either intravenous or oral doses, indicating that the formation of sulfation might be a rate-limiting factor to the bioavailability [175].

### Quercetin

#### Sources

The highest content of quercetin is in cranberries (1.49 g/kg), followed by yellow sweet savannah onions (650 mg/kg) [176].

#### Alleviating cardiac aging

As a potent SIRT1/PGC-1α activator [177], quercetin provides cardio-protection by reversing the effects of diabetes on SOD inhibition in the aged fatty rats [27], and improving aged-related cardiac dysfunction through activating PGC-1α, SOD, and mitochondrial function while reducing inflammatory markers in mice [28]. Direct evidence of quercetin on alleviating cardiac aging in humans is lacking, but the number and duration of ischemic episodes reliably decreased by using quercetin in patients with ischemic heart disease [178]. Importantly, the reduced vulnerability is evident in human cardiomyocytes incubated with quercetin under hypoxic condition, which is associated with quercetin-mediated activation of mitophagy in a SIRT1-dependent manner [179]. Thus, the ability of quercetin to restore myocardial cell viability through ameliorating mitophagy may be useful for alleviating cardiac aging in humans.

#### Alleviating CVD

Quercetin has the highest antioxidant capacity among plant polyphenols [180] to modulate the levels of different antioxidant enzymes, such as SOD and catalase [181, 182]. The protective effect of quercetin on CVD has been demonstrated in human studies [49, 183–185]. For example, daily treatment of quercetin (120 mg) for two months significantly improved LV diastolic/systolic function in patients with stable coronary heart disease [50]. Likewise, 150 mg of quercetin [51] improved blood pressure and reduced concentration of oxidized low density lipoprotein (LDL), a risk factor for CVD, in patients with hypertension or obesity [48, 51, 52]. Moreover, a 500 mg intake of quercetin for 70 d greatly reduced the blood pressure and plasma concentration of some inflammation markers, in women with type 2 diabetes [49].

#### Conundrum

Quercetin has low bioavailability, which is attributed to first-pass metabolism and being modified as methylated or dehydroxylated forms [186]. Quercetin can be administrated intraperitoneally or orally. Compared to oral administration, the intraperitoneal method has less effect on systemic blood pressure, which is attributed to a higher content of methylated metabolites of quercetin after taking it orally [187]. A clinical trial has reported that quercetin from rutin is more bioavailable in women than in men [188]. Co-administration with high-fat meals is suggested to improve the bioavailability of quercetin [189], indicating that quercetin requires a specific application form and dose for keeping its activity.

### Curcumin

#### Sources

It is an active constituent of turmeric with 31.4 g/kg of pure turmeric powder [190].

#### Alleviating cardiac aging

Curcumin promotes heart performance by improving VEGF-related angiogenesis in an old rat heart [29]. Although direct evidence of curcumin on retarding cardiac aging in humans is unavailable, a randomized clinical trial has demonstrated that curcuminoid treatment significantly decreases the incidence of in-hospital myocardial infarction, along with a reduced level of C-reactive protein, plasma malondialdehyde, and N-terminal pro–B-type natriuretic peptide in old patients [age (61 ± 9) years] after coronary artery bypass grafting [53]. Importantly, curcumin activates autophagy in vascular endothelial cells [191], which may be a useful property for alleviating cardiac aging in humans by protecting endothelial cells from oxidative stress.

#### Alleviating CVD

The effects of curcumin on preventing CVD in humans have been demonstrated through its antioxidant, anti-inflammatory, and lipid lowering properties. Administration of curcumin for 12 weeks caused a significant increase in total antioxidant capacity and a decrease in the expression of inflammatory markers in diabetic patients with coronary heart disease [54]. The lipid-lowing effect of curcumin is mainly reflected by reducing serum LDL, triglyceride [192], and free fatty acid in diabetic patients [55]. It is interesting that curcumin attenuates abnormalities of the vascular system [56] and LV afterload [57] preferentially in postmenopausal women. The magnitudes of the improved endothelial function with curcumin, as reflected by an increase in flow-mediated dilation, are comparable to that obtained with exercise in normotensive postmenopausal women [56], suggesting that curcumin treatment may be a special alternative approach against CVD in normotensive postmenopausal women who are unable to exercise. Regular aerobic exercise improves endothelial function in association with increased NO bioavailability [193]. It is unclear whether curcumin can improve endothelial function in men through a similar mechanism as in postmenopausal women.

#### Conundrum

Due to the relatively low absorption of the small intestine and fast metabolism, the oral bioavailability of curcumin is low [194]. However, it can be significantly increased by piperine through slowing the metabolism of curcumin [195]. It is evident that a combination of curcumin with piperine improves the lipid-modifying effect in patients with metabolic syndrome [196]. Studies showing no effect of curcumin on blood lipid profile were conducted with unformulated curcumin [197]*,* which, in contrast to curcumin prepared in amorphous forms [198] or nanoparticles [199], is considered to have low bioavailability.

### EGCG

#### Sources

EGCG is exclusively in tea, including green, black and oolong tea. Green tea has the highest levels of EGCG relative to oolong tea and black tea [200, 201].

#### Alleviating cardiac aging

The anti-cardiac aging effects of EGCG have been demonstrated by improving cardiac function and reducing LV hypertrophy in mice [30] and rats [31]. One of the mechanisms is associated with the antioxidant activity of EGCG, thereby reducing circulating lipids, such as LDL, triglyceride, and inhibiting ROS-related activation of inflammatory factors in the aged rat heart [31]. On the other hand, the mechanism is also attributable to the effect of EGCG on inhibiting histone deacetylase 1 and 3, thereby up-regulating the acetylation of cardiac troponin I in the aged mouse heart [30]. The question remains unclear as to whether EGCG alleviates cardiac aging in humans. However, emerging data have revealed that increased histone acetylation is a hallmark of aging [74, 202], and inhibition of which is beneficial in the heart by preventing oxidative stress and inflammation [203]. This may be associated with the role of EGCG in promoting autophagy-dependent survival [204], thereby protecting the heart against myocardial ischemia/reperfusion injury [205].

#### Alleviating CVD

EGCG prevents atherosclerosis and cardiac hypertrophy via anti-oxidant [206], anti-inflammatory [207], and lipid-modulating properties [208]. Regarding the effect in humans, Kuriyama et al. [209] have demonstrated that older adults consuming five or more cups of green tea daily can significantly prevent CVD-related mortality. This is further supported by two population-based studies in Greece demonstrating that only green tea displays an association with greater levels of physical activity and reduced likelihood of hypertension [210], of which, the mechanism is attributed to the high levels of EGCG in green tea [210]. This notion is further supported by clinical studies, in which EGCG can ameliorate endothelial dysfunction in patients with coronary artery disease [58] or early atherosclerosis [59], while also reducing LV myocardial mass in patients with wild-type transthyretin amyloid cardiomyopathy [211]. Thus, most of the pharmacological effects are attributed to its antioxidant properties. One hypothesis is that the EGCG molecule is a strong electron donor due to its eight hydroxyl groups, thereby possessing an efficient ability in scavenging ROS [212]. Of interest, EGCG has a sex-dependent effect on reducing total cholesterol in males and LDL-cholesterol in females [213]. However, the efficacy of EGCG in humans is inconsistent, and either positive [60] or not proven [214, 215] due to a major problem with low levels of stability and bioavailability.

#### Conundrum

The bioavailability of EGCG is rather low (i.e., 0.2–2% of total ingestion) in humans under healthy conditions [216], but increases linearly with the dose of each intake [217]. However, patients have shown liver toxicity after continuous daily intake of more than 800 mg of EGCG of green tea [218]. Recent study has demonstrated that oxidized phosphatidylcholines can specifically deliver EGCG to intimal macrophages through cluster of differentiation 36 (CD36) receptors, while reducing toxicity due to the accumulation of EGCG in the liver [219]. This molecular approach has suggested a potential clinical usage by using biocompatible and biodegradable ligand-coated-EGCG nanoparticles to atherosclerosis formation.

### Spermidine

#### Sources

Spermidine can be derived endogenously from amino acid catabolism, such as arginine, and presented in almost all eukaryotic and prokaryotic cells in millimolar concentration [220]. However, dietary spermidine is the major source of polyamines in vivo [221]. Spermidine is present in many foods, with comparable amount (100–200 µmol/kg) in vegetables, fermented cheeses, and meats, while less than 50 µmol/kg in bread, potatoes and fruits [222].

#### Alleviating cardiac aging

Oral supplementation of spermidine can alleviate cardiac aging in mice [32, 33] and rats [32, 35] by enhancing autophagy, mitophagy [32] and mitochondrial respiration [223]. Spermidine also reverses arteria aging in mice by restoring NO-related endothelium-dependent dilation and reducing oxidative stress in an autophagy-dependent manner [34].

#### Alleviating CVD

Spermidine has become a leading candidate for treating CVD in humans [224], by promoting autophagy and mitophagy, as well as reducing systemic chronic inflammatory responses [84] and mortality [225]. From a clinical perspective, hypertension occurs in most elderly patients suffering from CVD [32, 226]. High levels of dietary spermidine correlate with reduced blood pressure and low incidence of CVD [32] through inhibiting pro-inflammatory status in humans [61]. Spermidine intake has been observed to be greater in women than in men, and declines with age [225]. The regression analysis showed that plasma levels of spermidine tended to change with age [227] and sex [228]. However, due to large individual differences, trends were not statistically significant [228]. In fact, the exact biological mechanisms underlying the individual differences remain unknown. A good tolerability with no safety concerns is reported post supplementation of spermidine-rich plant extracts with older adults [229]. In addition, spermidine has neither adverse effects on glucose and insulin metabolism [32], nor side effects. Thus, keeping an optimal level of the polyamine content in various organs is an important consideration in the elderly because the activity of ornithine decarboxylase, a key polyamine biosynthetic enzyme, decreases with age [230].

#### Conundrum

The exact molecular mechanisms responsible for spermidine-induced activation of autophagy in cardiomyocytes remain unknown because the internalization or uptake of exogenous spermidine by cardiomyocytes has not been investigated. Based on the observation that the level of cardiac spermidine is higher in mice post spermidine treatment, it has been proposed that the exogenous spermidine is preferentially taken-up by cardiomyocytes [32]. Thus, it is necessary to establish in vivo studies to investigate the underlying mechanisms of spermidine uptake by cardiomyocytes. This may help us to understand whether it is the differences in dosage or routes of administration that have caused inconsistent findings.

## Perspective

### What remains unclear?

On the mechanistic level, the above-mentioned CR-mimetics protect the heart and blood vessels through reducing mitochondrial damage, apoptosis, inflammation, and oxidative stress, which are relevant to delay cardiac aging and are associated with autophagy activation [147] via improving mitochondrial turnover. However, an increased autophagy is evident in a mouse heart with type 1 diabetes, while a decrease of which is seen in type 2 diabetes [231]. Likewise, the numbers of autophagosomes are increased in cardiac tissues of patients with LV-hypertrophy [232], aortic valve stenosis [233], hibernating myocardium [234], and HF [235]. What are the responses of autophagy to cardiac function in these conditions: adaptive or maladaptive? It has been proposed that low levels of autophagy can protect cardiomyocytes and endothelial cells, whereas excessive of which do the opposite via damaging mechanisms [236]. To date, there is no available method to assess autophagy flux within the human body or specific organs, which challenges the investigations to identify the optimal autophagic activation windows. Hence, the current clinical evidence is insufficient to discuss if their cardiac effects are systemic or organ specific. Another puzzle is that the beneficial effects on CVD in humans are inconsistent, and inequitable to the dosing of CR-mimetics. Hence, should CR-mimetics be a supplemental molecule that can boost the effectiveness of existing medical drugs for CVD? From a dietary perspective, the main challenge of alleviating cardiac aging is to quantify the degree of prevention achieved in healthy individuals. Thus, what are the most relevant features to be evaluated? Collectively, to understand and quantify the therapeutic efficacy of CR-mimetics in a human heart, we propose that a few factors should be taken into consideration.

### What more should be considered?

First, a dose-dependent response and long-term observation with higher sample size. Most of the interventional studies with the above-mentioned CR-mimetics lack the dose response relationship with long term effects. They have focused on traditional cardiovascular risk factors, such as obesity, hypertension, and hyperlipidemia [32, 223, 237], without considering the cardiovascular endpoints, such as age-related atrial wall stiffness and flow-mediated dilation. In addition, bioavailability and metabolization are important factors for designing clinical experiments and ensuring therapeutic efficacy. Low oral bioavailability may be a major challenge associated with the ambiguous therapeutic effects in clinical trials, from positive [42, 44] to ineffectual [238–240] or large individual variations [228]. Factors causing low bioavailability may include not only their content in the diet and intake in humans, but also their metabolic processes mediated by the liver, intestines and microbiota [241]. As an example, dietary intake levels of total flavanols are estimated to vary greatly from 386 mg/d in Germany [242], 192 mg/d in the United States [243], and 23 mg/d in the Netherlands [244]. Likewise, daily intake of spermidine is reported to correlate with gross domestic product [245]. These discrepancies cause problems for estimating an efficient intake, which certainly influence dosing schemes and clinical outcomes. With respect to absorption, the forms displaying in circulation are often not the forms seen in food, indicating that absorption is accompanied by certain conjugation and metabolism. As an example, quercetin in a form of glucoside in onions is efficiently absorbed and bioavailable compared to the free forms [246] or other forms containing rutin in apples and tea [247]. In contrast, plasma EGCG is mostly in an unconjugated form, and has proven to have higher activity than its metabolites [248]. Lastly, a combinational therapy should be considered. Biological cardiac aging is a multifactorial process with many systemic contributing mechanisms occurring within the heart and surrounding tissues. As such, it is unlikely that any single pathway or intervention would fully restore age-related cardiac phenotypes. It has been demonstrated that the impact of combinational therapies on boosting the functional abilities of vasculature and endothelial function is greater than those of individual treatments in postmenopausal women [249, 250]. Based on the observation that curcumin pharmacokinetics are affected by sex [251], we propose that the dosage of polyphenol or polyamine, personalized as a “one‐dose‐fits‐all” strategy, is unlikely to work. Thus, as an example of combinational therapies, we propose that stimulating glucose oxidation, by pyruvate dehydrogenase kinase inhibitor [252], along with a supplementation of polyphenol or polyamine, may be beneficial to human aging hearts [253]. The concept is that glycolysis is increased in the aged heart along with unaltered glucose oxidation [14, 254]. As a result, glycolytic intermediates are diverted to form advanced glycation end-products [255] (Fig. 1b), which can be recognized by CD36 [256] causing oxidative stress-related inflammation and endothelial dysfunction [257, 258]. Thus, stimulating glucose oxidation will amplify the effect of CR-mimetics by enhancing ATP production in concert with improving systemic glucose tolerance and inflammation. This notion can be further supported by others showing that a combination of spermidine from a natural product with nicotinamide inhibits oxidative stress and improves endothelial cell survival, in a potentiated manner than each compound alone, in platelets isolated from patients with metabolic syndrome [259]. This also holds true for the effect of combinational resveratrol-piperine with α-tocopherol (has an anti-inflammatory activity [260]), in a clinical trial [47], in which, the decrease in arterial hypertension and inflammation is evident in older patients [age (68 ± 4.7) years] with metabolic syndrome [47].

## Conclusions

CR-mimetics show promising results in alleviating aspects of CVD in humans. Based on the role of autophagy in the prevention of age-related conditions, CR-mimetics can be considered as a potential therapeutic tool for alleviating cardiac aging in humans, which is especially true for cardiomyocytes due to the heavy reliance on mitochondrial oxidative metabolism to keep contractility. However, the baseline intake of dietary CR-mimetics will influence the outcomes of dosing schemes. Sex-specific differences in absorption, distribution or excretion of the interventional compounds have important clinical consequences and side effects, which may affect the therapeutic efficacy and anti-aging potential in humans. To improve the clinical effectiveness, some important data need to be addressed from CR-mimetics, such as bioactive levels, oral bioavailability, metabolization, organ-specific effects, and interaction with body-endogenous biosynthesis pathways. Larger size of participants and longer follow-up are required to confirm the clinical effect.

## Data Availability

Not applicable.

## Abbreviations

ATP: Adenosine triphosphate
CD36: Cluster of differentiation 36
CR: Caloric restriction
CVD: Cardiovascular disease
eNOS: Endothelial NO synthase
EGCG: Epigallocatechin gallate
ERs: Estrogen receptors
HF: Heart failure
HFpEF: HF patients with preserved ejection fraction
HFrEF: HF patients with reduced ejection fraction
LDL: Low density lipoprotein
LV: Left ventricle
NAD: Nicotinamide adenine dinucleotide
NO: Nitric oxide
PGC1α: Peroxisome proliferator-activated receptor gamma coactivator 1-alpha
ROS: Reactive oxygen species
SIRT1: Sirtuin 1
SOD: Superoxide dismutase
VEGF: Vascular endothelial growth factor
